# Mobile application development for improving medication safety in tuberculosis patients: A quasi-experimental study protocol

**DOI:** 10.1371/journal.pone.0272616

**Published:** 2022-09-07

**Authors:** Erlina Wijayanti, Adang Bachtiar, Anhari Achadi, Ummi Azizah Rachmawati, Amal Chalik Sjaaf, Tris Eryando, Besral, Trihono, Kemal N. Siregar, Dhanasari Vidiawati

**Affiliations:** 1 Faculty of Public Health, Universitas Indonesia, Depok, Indonesia; 2 Faculty of Medicine, YARSI University, Jakarta, Indonesia; 3 Faculty of Information Technology, YARSI University, Jakarta, Indonesia; 4 Ministry of Health, Jakarta, Indonesia; 5 Faculty of Medicine, Universitas Indonesia, Jakarta, Indonesia; Flinders University, AUSTRALIA

## Abstract

The COVID-19 pandemic, the growth of smartphones, and the internet have driven the use of technology for monitoring TB patients. Innovation in management of TB patients is needed to improve treatment outcomes. The study was conducted to obtain a predictive model of medication safety and solution model for at-risk patients, and to improve medication safety through mobile applications. The research was conducted in 4 stages, namely qualitative, quantitative (cross-sectional), qualitative, and quantitative (quasi-experimental, post-test group control design). Data were taken at the Public Health Center in Jakarta, Indonesia. Samples were taken by cluster random sampling. For quantitative research, 2^nd^ phase (n = 114) and 4^th^ phase (n = 96) were analyzed using logistic regression. This study analyzed predictors of medication safety to assist in monitoring patients undergoing treatment. At-risk patients were educated using an algorithm programmed in the application.

## Introduction

Tuberculosis is one of the world’s top 10 causes of death [1]. In 2020, there were 845,000 estimated TB cases with 543,874 notified, 35% of cases unreported, and a treatment success rate of 84%. Cases of Multi-Drug Resistant TB increase annually, with 9,038 recorded in 2018 and 10,097 in 2019 [2]. Treatment failure results in drug resistance, leading to expense, lower cure rates, severe side effects, and longer treatment [3].

The length and complexity of TB therapy affects patient compliance. The most common TB drug therapy problems are adverse reactions (62.3%) and non-adherence (21.5%) which can be solved by therapeutic monitoring [4, 5]. Adverse drug reactions cause non-adherence and drug resistance [6].

Under pandemic conditions services must prioritize patient safety, respond to COVID-19 and to keep TB services running [5, 7]. Evaluation of a TB program in Indonesia during the pandemic showed medication nonadherence, patients not collecting sputum, cessation of referral laboratory examinations, and issues monitoring patient compliance [8].

The WHO recommends using technology for drug ingestion control. Remote patient monitoring is rapidly increasing while 75.6%, of Indonesia’s population uses mobile phones. Up to 47.7%—58.6% urban, 33.8% rural, and 76.2% of Jakarta’s population—use the internet [9].

Studies suggest adherence interventions for improving outcomes, such as medication event reminder monitor (MERM) systems, electronic pillboxes, and text messaging [10, 11]. Medication reminders appear to improve compliance and staff-patient relationships, but have not been shown to cause significant differences in treatment success [12].

In one study medication monitors (MMs) reduced missed doses [13]. In another study, patients using an electronic medication monitor (EMM) displayed no significant differences from patients without EMM [14]. More research is needed to assess effects of technology and intervention on TB programs [15].

Indonesia’s Ministry of Health has developed applications such as SOBAT TB, which focus on independent TB screening, finding health facilities, and connecting the TB patient community. EMPATI-TB monitors and assists treatment of drug-resistant TB, while EMPATI CLIENT monitors patients’ medication use via video, allowing patients to view medication history and consult with cadres or companions [16, 17]. Meanwhile, the Tuberculosis Information System (SITB) is a web-based platform used by officers to report patients in treatment. Each application has advantages, but none uses a predictive model to prevent non-compliance.

The COVID-19 Pandemic has revealed the necessity of made application that remotely detects risks and monitors primary-care tuberculosis treatment.

### Research questions

What are predictors of medication safety for primary-care tuberculosis patients?

How can an algorithm monitoring medication safety function within a mobile application?

Can mobile applications influence medication safety?

#### Objectives

Assessing effects of mobile applications on tuberculosis medication safety.

Specific objectives.

- Knowing medication safety predictors in primary-care tuberculosis patients
- Crafting a decision-making model for at-risk patients

## Materials and methods

**Fig 1:**
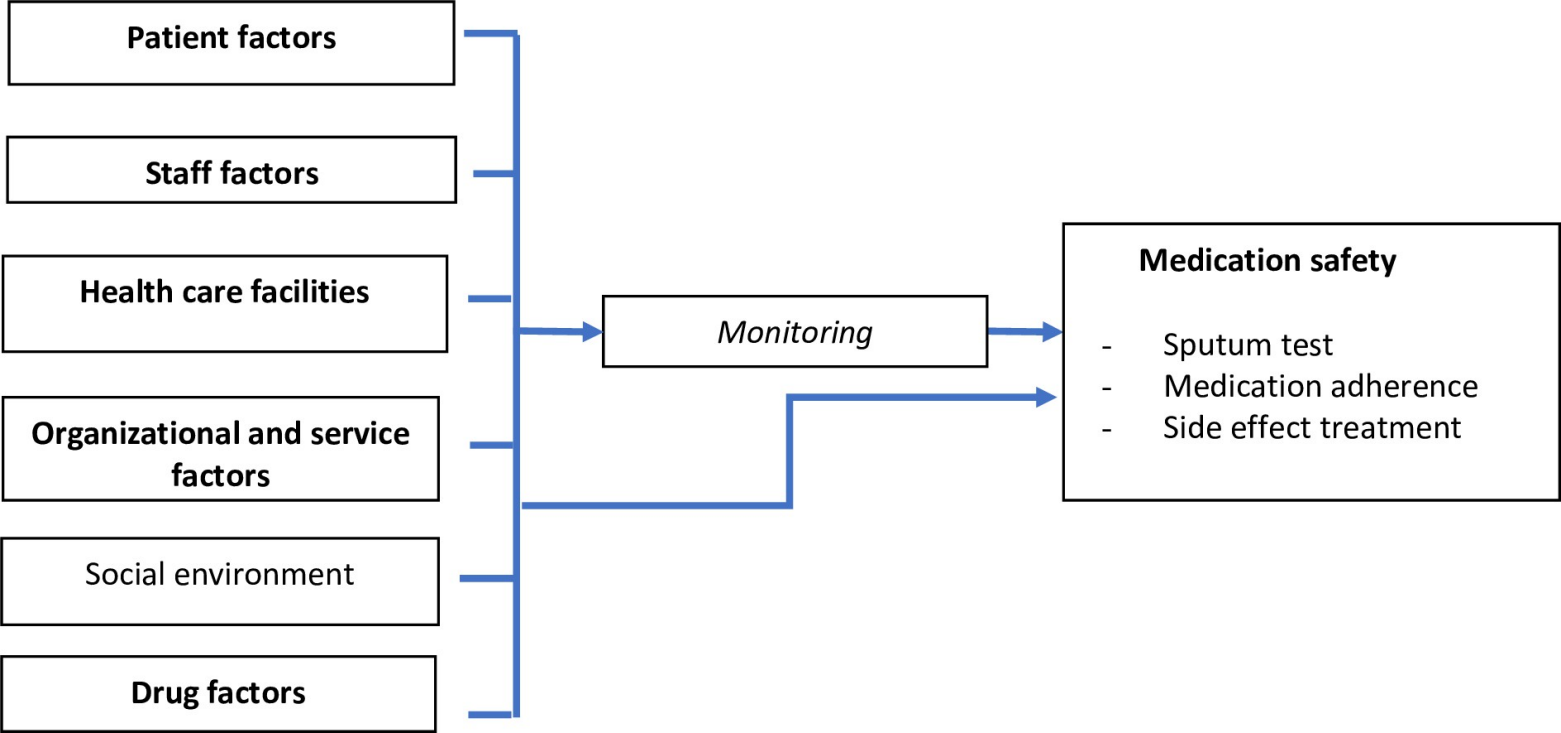
Conceptual framework (1st, 2nd, and 3rd phase of the study).

**Fig 2:**
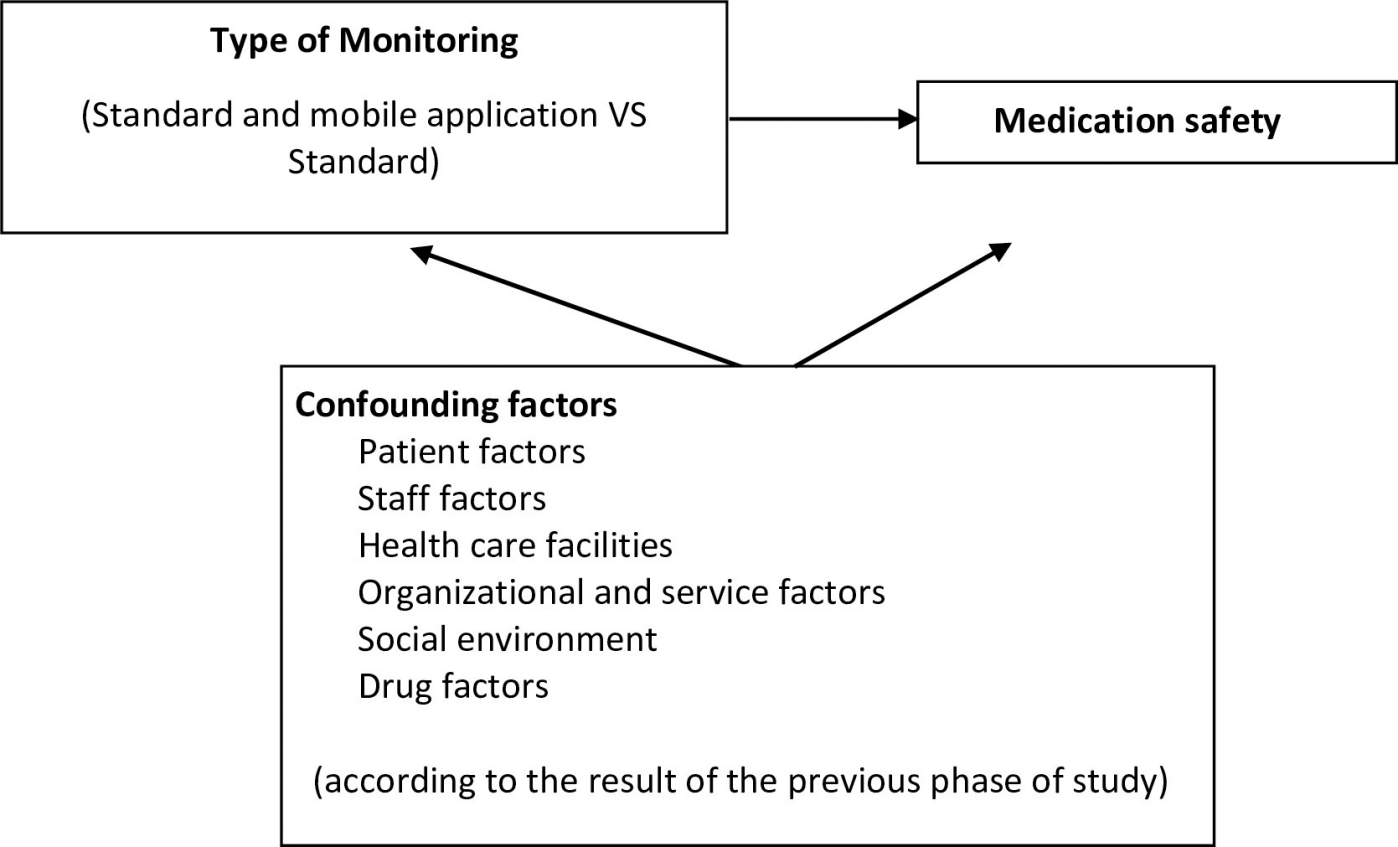
Conceptual framework (4th phase of the study).

**Fig 3:**
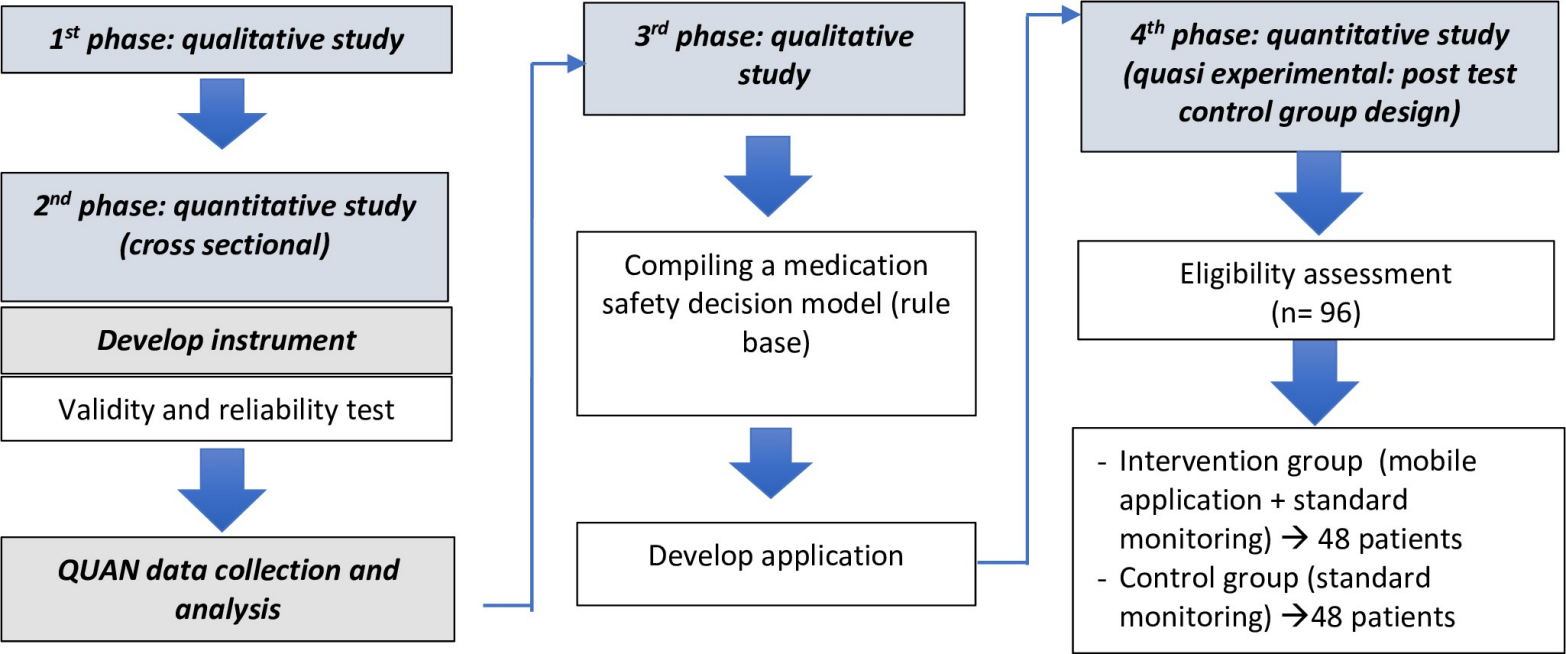
Research stages.

The conceptual framework of the research can be seen in Fig 1, which describes research phases 1, 2, and 3. Phase 4 is illustrated in Fig 2. These objectives were addressed using operational research, carried out sequentially in 4 stages (Fig 3) in June-December 2021. Details of each stage can be seen in Table 1. Table 2 describes the flow of quasi-experimental research (4^th^ stage).

**Table 1:** The description of study.

Description	Phase of study			
	1^st^	2^nd^	3^rd^	4^th^
Output	Exploration of independent variable (medication safety)	Medication safety model	Decision-making model for improving medication safety	Effect of application on medication safety
Collecting data	In-depth interview	Online questionnaire (optional telephone assistance)	In-depth interview	Interview and observation (using application)
Subject	TB manager at public health center, patient, expert, and policymaker	Inclusion criteria:	TB manager at public health center, cadre, patient, expert, and policymaker	Inclusion criteria:
		New pulmonary TB patients, 18–65 years old, who have undergone initial phase of treatment (category I)		New patients with bacteriologically confirmed or clinically diagnosed pulmonary TB, starting treatment (category I), 18–65 years old, have regular access to a smartphone and an internet connection, can operate a telephone or have someone to help.
		Exclusion criteria:		
		Serious side effects, difficulty communicating, HIV (+)		
				Exclusion criteria:
				Patients sharing household with other subjects, HIV (+), drug-resistant TB, serious side effects, difficulty communicating
Sampling	Purposive sampling	Cluster random sampling	Purposive sampling	Cluster random sampling
Number of samples	13 respondents	Minimum required sample used formula of two population proportion two side, significant level = 5%; power test = 80%, P1 = 80.2%, and P2 = 54.2%.	15 respondents	Minimum required sample used formula of two population proportion two side, significant level = 5%; power test = 80%, P1 = 61%, and P2 = 31%.
				P1 was adherence in initial 2 months of treatment in group using Video Observed Therapy, while P2 was adherence in group without Video Observed Therapy [19].
		p1 was proportion of adherence in patients supported by family. p2 was adherence in patients not supported by family [18]. Number of samples required was 57 per group or a total of 114 people (with 10% dropout anticipation)		
				Number of samples required was 48 per group or a total of 96 people (with 10% dropout anticipation)
Analysis	Transcribing, checking, coding, theming	Logistic regression	Transcribing, checking, coding, theming	Logistic regression

**Table 2:** The flow of the quasi-experimental study.

Before therapy				Two months of initial treatment	After 2 months of treatment
Passive case finding	Diagnosis	The education and counseling related to TB treatment	Requesting the consent from patients to be involved in research	The observation and intervention for 2 months	Collecting data, including predictors and medication safety by interviewing, viewing compliance record cards, and viewing medication safety data from the application (in the intervention group)
Active case finding		The assessment of patient eligibility			

The informants involved in qualitative study consisted of various levels. Respondents were selected with various characteristics to enrich the information. Officers were diverse, representing different types of public health centers (sub-district/ward) and profession (general practitioner/nurse). Patients too were diverse, representing compliance 100% or under 100%, experienced the side effects (yes or no), and had families/other than families serve as drug swallowing supervisors. Data validation was carried out by theoretical triangulation. This sampling design is expected to describe the condition in Jakarta.

**Fig 4:**
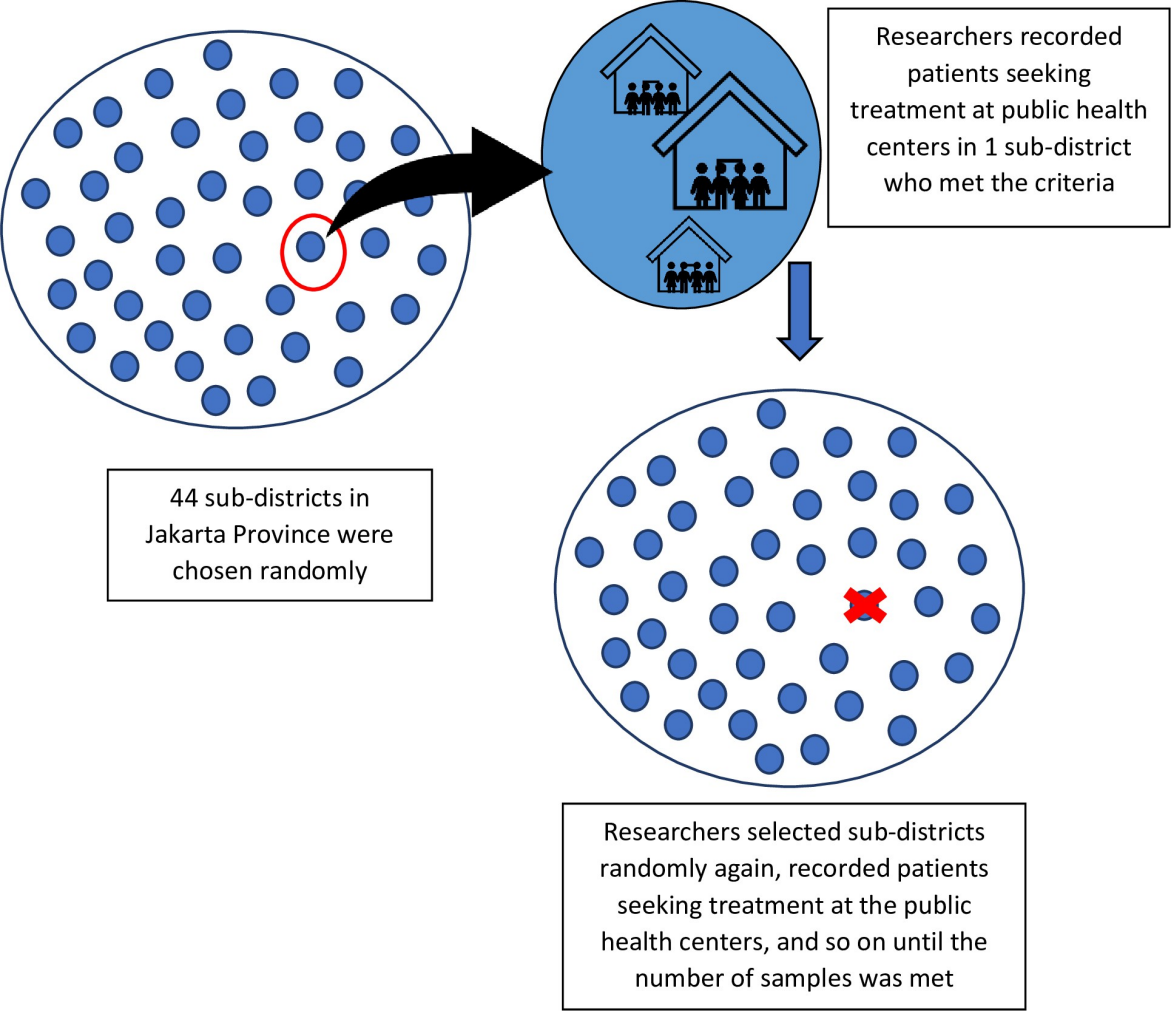
Cluster random sampling based on sub-district.

Cadres were involved in the 3rd phase of this study because they could provide additional information regarding the findings of the 2nd phase. Cadres were more aware of the technical conditions in the field, such as the condition of the patients’ families and environmental conditions around them. Besides, they could play a role in reducing the stigma on patients, families, and communities. Cadres could also function as companions while patients were undergoing treatment and carry out contact investigations to track suspected patients until they were controlled and recovered. Fig 4 illustrates sampling in quantitative research.

### Operational definition

The medication safety assessment included monitoring sputum examination, medication adherence, and side effects. It was measured by interview. Intervention group data was recorded in the application. Failure to conduct a repeat sputum examination, medication compliance at <100%, and mishandled side effects led to risk.

### Intervention protocol

Subjects were divided in 2. The intervention group received standard monitoring plus application usage. The control group received standard monitoring. Intervention was carried out for 2 months following start of treatment. Data was collected through questionnaires completed in the application (intervention group) or as Google forms (control group), at the study’s beginning and end.

The effect of applications were measured for 2 months. Early intervention is key, with germs decreasing effectively in the first 2 months of treatment. In this phase, patients often experience drug side effects [20].

Validity and reliability tests were carried out with a 35-person questionnaire. Data analysis was performed by bivariate test. Variables with p value < 0.25 were included in the multivariate test using logistic regression. Variables with p value < 0.05 in multivariate analysis were referred to as predictors. Insignificant variables (p > = 0.05) were excluded. A variable causing a > 10% change in odds ratio of other variables was included and became a confounder.

The application developed is called ERLINA (e-Empowerment Resource for Lowering Ignorance and Negligence Action in therapy) and is free to download through the Android playstore.

**Fig 5:**
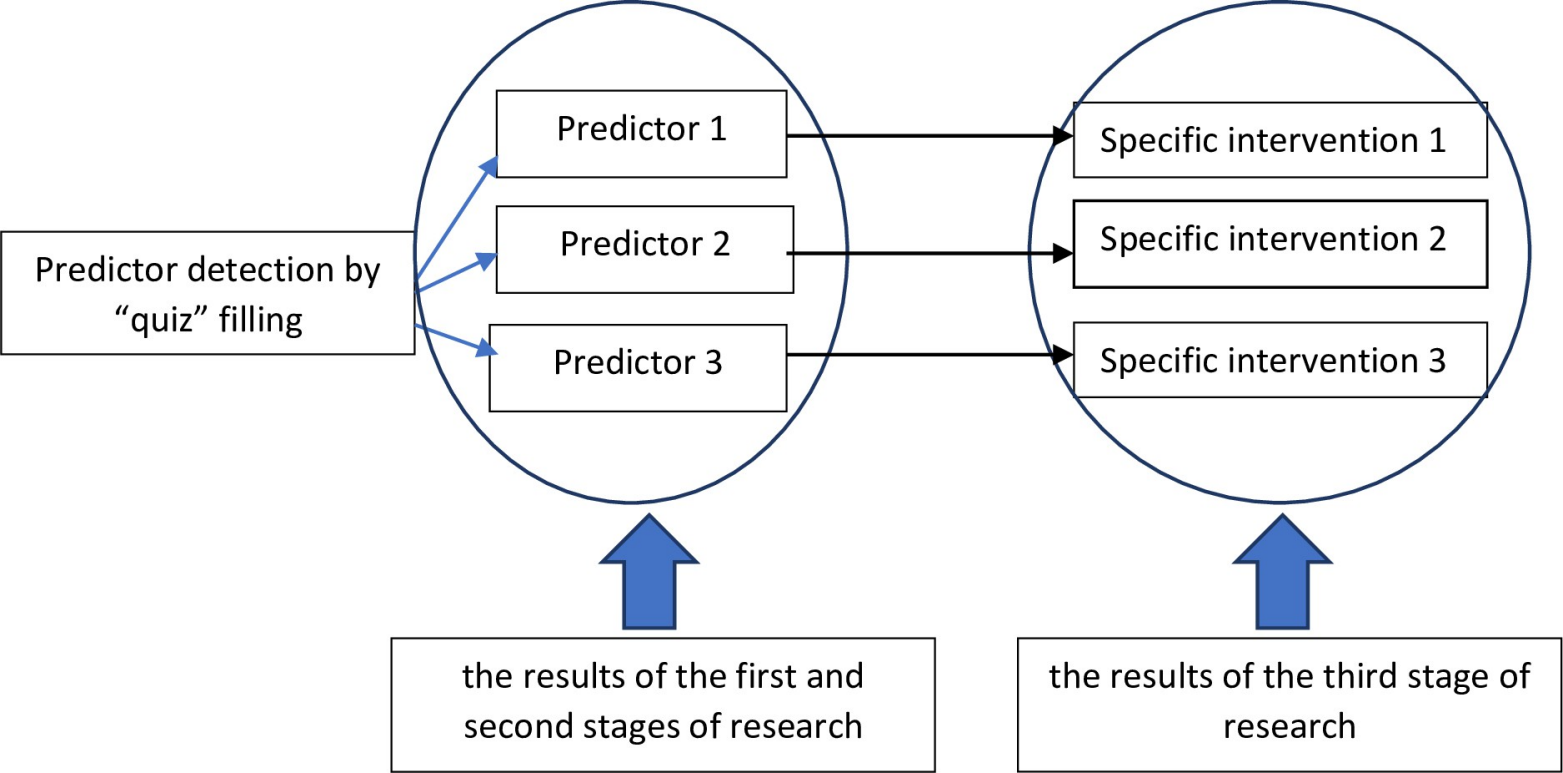
Development of specific intervention algorithm.

**Fig 6:**
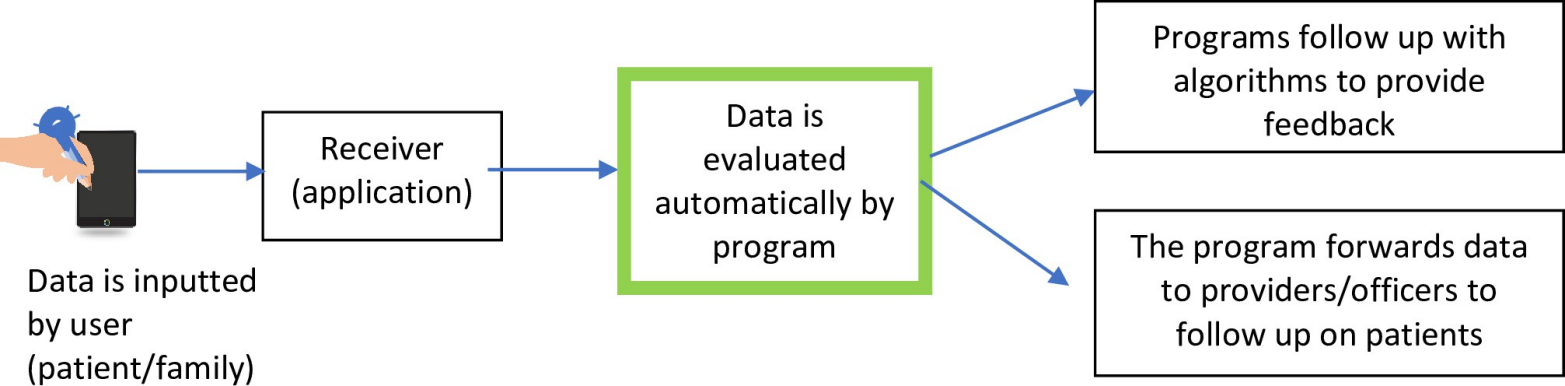
Integration of remote patient monitoring and decision support system.

A decision support system algorithm requires identifying predictors of medication safety alongside specific intervention models (Figs 5 and 6). The application was tested by IT experts and doctors, who assessed the system’s functioning and content.

**Fig 7:**
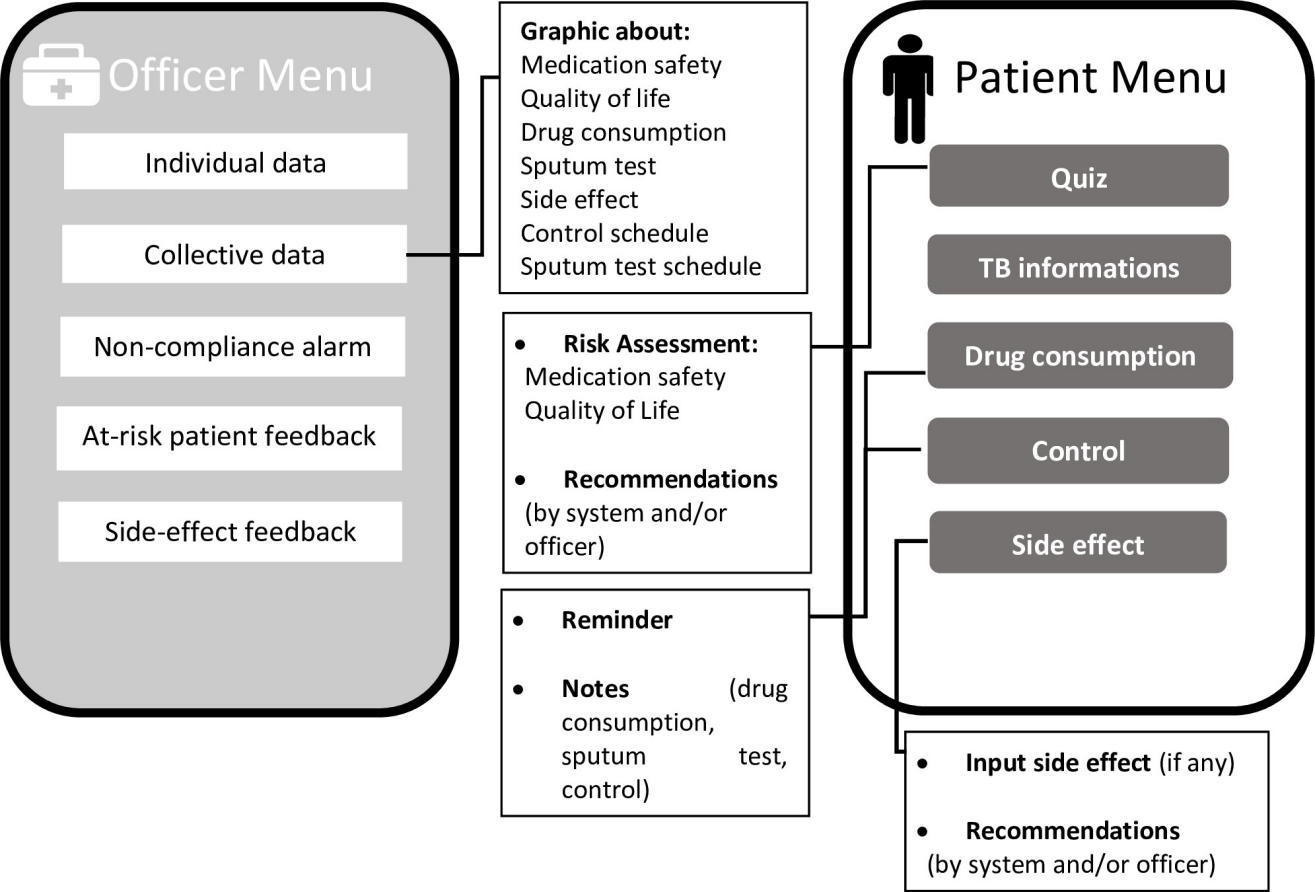
User interface design of the application.

The application’s functions predict medication safety risks and provide reminders and education (Fig 7). Dashboards allow officers to monitor and provide feedback. Patients input medication, phlegm, and control data when the reminder sounds. Data is collected in the patient compliance chart visible to staff. Patients who miss > 6 doses are included in a non-compliance list in the staff menu. Side effects are reported by the patient to the officer and are automatically addressed by the application and staff.

### Potential obstacles include

1. *Varied patient absorption of information*. Audio-visual media, straightforward language, and officer assistance help ameliorate this.
2. *Real-time data monitoring without internet*. In the absence of internet, a revision menu is provided, letting patients change data when they have internet access. Patient data is checked with the regularity of taking medication on the control card (TB01 card).
3. *Lack of validation*. Information content may not follow Public Health Center standards. Officers were involved in developing applications, especially in qualitative research stages 1 and 3. Public Health Center doctors helped develop validation tests to ensure the application could support TB services.
4. *Data privacy*. All data entered by the patient, excepting passwords, is visible to officers. At the start of the study and during registration, patients consented to data monitoring.

### Ethical review

Before data collection, researchers offered information about procedures. Participants consented via electronic questionnaire. In in-depth interviews, verbal consent was recorded. This research has passed the ethical review by the Committee for Research Ethics and Community Service, Faculty of Public Health, University of Indonesia (no 79/UN2.F10.D11/PPM.00.02/2021) on March 22, 2021.

## Results and discussion

Data from the first phase of the research is presented (Table 3) as a basis for developing a conceptual framework.

**Table 3:** The results of the first stage of research (predictors of medication safety).

Predictor	Component	Information
Patient	Characteristics	• Age
		• Gender
		• Income
		• Working patients
		• Patient education
		• Patients a lot of activity
		• Comorbid
		• Out-of-town patients
		• Patients move health facilities
		• Patients moving house
		• The patient does not have a cellphone
	Perceptions	• Patient commitment
		• Motivation to heal
		• The patient already feels well
		• Positive thinking
		• The patient does not feel alone
		• Patient perception of treatment
		• Patients who feel heavy with prolonged treatment
		• Reception of patients with the disease
		• Shame on treatment
		• Boredom of the patient in taking medications
	Habits	• Use alarms/reminders
		• The habit of recording schedules
		• Regular waking habits
	Personalities	• Patients forget easily
		• Activeness of the patient for consultation
		• The patient’s ability to cope with emotions to stay calm
	Patient knowledge	• the patient cannot distinguish between side effects and symptoms that are not from the side effects of the drug
		• Patient understanding of the impact of irregular treatment
	How to get to the health facility	• Required transportation costs
		• Distance from the patient’s home to the health facility
	Related to treatment	• The patient finds it difficult to take the medicine
		• Patients have difficulty expectorate sputum
		• Adaptability of patients in undergoing treatment
Social	Family	• Family responsibility to support patients
		• There is a drug swallowing supervisor that assists the patient
		• Drug swallowing supervisor who understands medicine
		• Family support
		• Awareness of people around the patient
	Community	• Support from friends
		• The role of supportive cadres
		• Stigma and discrimination
		• Community support
		• There is a specific group or community of patients
Officer	Performance	• Discipline of officers in serving TB patients
		• Monitoring of taking medications virtually
		• Officers with double duty
	Communication	• Describe treatment plans, the importance of TB treatment, and side effects
		• Make patient commitments at the beginning of treatment
		• Ease of access to healthcare workers
		• Officers ask for side effects
		• Counseling
		• The attendant reminds the patient
		• Effective communication for TB education
		• Effective communication media
		• Providing motivation to patients
		• Providing information to the drug swallowing supervisor
	Interaction	• Building patient and attendant trust
		• Emotional support from officers
		• Rewarding patients who comply
		• A family approach
Healthcare		• TB services that make it easier for patients
		• Use of tuberculosis information system to view the patient’s therapy schedule
		• There is a card that records the schedule of control and taking medications
		• Laboratory facilities
		• Access to standard laboratories
		• Application to monitor patients
Organizational culture		• Risk management
		• Continuous quality evaluation
Medicine		• Drug availability
		• Taking TB medications take a long time
		• The presence of side effects of the drug
		• Information on how to take medication
External		• Coordinate with local citizens
		• The existence of TB program monitoring at various levels

The variables from the data above were selected and developed into operational definitions. The operational definition (Table 4) details helped compose the questionnaire. Grouping variables based on percentiles divides the data equally in each group.

**Table 4:** Operational definitions and measurements scales.

Variable	Definition	Likert scale	Measurement result and code
Medication safety	The condition of being free from injury or potential injury due to errors in the process of using drugs [21, 22]. The assessment includes the presence or absence of incidents in the monitoring process, namely monitoring the progress of treatment through sputum tests at the end of initial treatment, the medication adherence, and the handling of drug side effects [23].	No	0 = unsafe (if a patient fails to check sputum, medication adherence is < 100%, or side effects are not reported to the officer and treated adequately)
			1 = safe
Monitoring	Drug Swallowing Supervisor who are close to the patients and voluntarily want to be involved in assisting the treatment of them until they recover [24]	No	1 = family
			0 = other than family
**Patient factors**			
a. Age	The length of time that is counted since the patients were born	No	Age is classified into 4 categories according to the <25th, <50th, <75^th^, and ≥ 75^th^ percentiles.
b. Education	The formal education that has been completed	No	0 = no school/elementary school
			1 = junior-senior high school
			2 = diploma/bachelor
c. Knowledge	The knowledge that TB treatment takes a minimum of 6 months, the dose must be taken per day, sputum examination is repeated, and there are side effects of TB drugs [25]	No	0 = very poor (<25^th^ percentile)
			1 = less (<50^th^ percentile)
			2 = enough (<75^th^ percentile)
			3 = good (≥75^th^ percentile)
d. Perception	The patients’ response to TB, including the perceptions of the benefits of treatment, the barriers, the importance of therapy, and the self-efficacy [26]	Yes	0 = bad (<25^th^ percentile)
			1 = enough (<50^th^ percentile)
e. Habits	The daily habits of the patients in carrying out activities such as sleeping, eating, and taking TB medications regularly [27]	Yes	2 = good (<75^th^ percentile)
			3 = very good (≥75^th^ percentile)
f. Personality	The intrinsic traits that are reflected in the patients’ attitude, including setting targets, actively increasing knowledge, and asking questions about illness [27]	Yes	
g. Difficulty reaching health facilities	The barriers traversed by the patients to reach health facilities, including distance, transportation costs [28], and limited transportation due to the outbreak conditions	No	0 = difficult (if there are obstacles)
			1 = easy (if there are no obstacles)
h. Alcohol user	The patients’ habit of drinking alcohol [29]	No	0 = yes
			1 = no
i. Smoking	The patients’ habit of smoking cigarettes [29]	No	0 = smoker
			1 = non-smoker
j. Income	The patients’ income obtained from business or work	No	1 = high if above the Provincial Minimum Wage (> Rp4,416,187)
			0 = low if below or equal to the Provincial Minimum Wage (≤ Rp4,416,187) [30]
k. Comorbidity	The presence of other chronic diseases besides TB [31]	No	0 = yes
			1 = no
l. Traveling	The traveling out of town made by the patients while they were undergoing TB treatment	No	0 = yes
			1 = no
m. Distance from house to health facility	The distance from the patients’ houses to health facilities	No	1 = < 1km
			0 = ≥ 1km
n. Time to reach health facility	The time it takes for the patients to reach health facilities	No	1 = < 15 minutes
			0 = ≥ 15 minutes
**Staff factors**			
a. Performance	The performance of the officers, including respecting patients, involving patients in making decisions, showing empathy, and mastering TB care [27]	Yes	0 = bad (<25^th^ percentile)
b. Communication	The process of delivering information from the officers to the patients, including patients’ condition, TB disease, how to take TB drugs, side effects, encouragement to patients to believe that TB can be cured, and schedule of repeated sputum checks [27]	Yes	
			1 = enough (<50^th^ percentile)
			2 = good (<75^th^ percentile)
			3 = very good (≥75^th^ percentile)
c. Staff-patient interaction	The relationship between the officers and patients, including patient trust, sufficient time provided by officers to patients for talk, and good response to them [29]	Yes	
**Health care factors (facilities, organizational, and service)**			
a. Remote services	The services without direct care that use information and communication technology such as schedule control reminders, online registration, remote monitoring, and consultations	No	1 = existent
			0 = none
b. Access	The ease of getting services, including appointment and service flow [29]	Yes	0 = bad (<25^th^ percentile)
c. Patient safety culture	The organizational culture, namely characteristics and norms originating from within the organization [32] related to patient safety culture in this case, e.g. officers ensuring that patients understand the information provided, prioritizing patient safety in service, and motivating them to report errors while taking medication	Yes	
d. Drug services	The activities to meet patient needs related to free TB drugs [20, 27]	Yes	
e. Quality of service	The quality of service is characterized by patient satisfaction [25], including the attitude of the officers, the fulfillment of the patients’ therapeutic needs, and the impression of the building and layout	Yes	1 = enough (<50^th^ percentile)
			2 = good (<75^th^ percentile)
			3 = very good (≥75^th^ percentile)
Family support	The assistance (physical or moral) provided to TB patients, including reminding them to seek treatment and giving spiritual encouragement [33]	Yes	
Treatment	The perception of the effect of the drugs on recovery, the patients’ ability to deal with the side effects, and the difficulty in swallowing the drugs [29]	Yes	

Factors influencing medication safety include patient factors (demographics, social and family situation, comorbidities); staff factors (habits, patient relationships, social and medical conditions); health care facilities (infrastructure, access, information systems, remote services); organizational and service factors (protocol, organizational culture, workload, pharmacy service); external factors (physical environment, social environment, technology, infrastructure, policies); drug factors (effectiveness, side effects, patient adjustment to the regimen); and process (prescription, preparation, checking, storage, administering, information delivery, monitoring) [29, 34].

Adequate treatment involves the right combination of drugs at the correct dosage, taken regularly and monitored for a sufficient duration [20]. Patient education consists of which drugs to take, the amount and manner of doing so, the possibility of adverse reactions, the right time to seek treatment, consequences of not taking medication properly, and prevention of TB transmission [35].

Medication safety involves preventing or repairing drug use injury [36]. Medication safety outcome indicators include medication errors, incident type, and impact [37].

Areas monitored include response to therapy, regularity of taking medication, and drug tolerance, including adverse drug reactions [38]. Digital interventions for medication safety are important given pandemic-induced service limitations. Predictions are expected to reduce medication errors, while the Decision Support System (DSS) [39], supports but does not replace decision-makers [40]. Performing predictive maintenance involves observing conditions and making timely diagnoses before failure occurs [41].

Remote monitoring collects patient data using technology [42]. Benefits include rapid detection, continuous monitoring, reduced costs, daily patient information, increased health/emergency service efficiency, and assistance for limited-mobility patients [43].

### Limitations

- The research design did not use a randomized controlled trial.
- Stage 4 patients required cell phones and internet.
- The medication safety model, implemented in a mobile application, (measured in stages 1,2, and 3) involved a wide range of patients. In limited-equipment conditions, cadres helped ensure safety.
- Research has not measured impacts such as treatment success.
- The application was unintegrated with the tuberculosis recording system (SITB): patients enter medication schedules and checked sputum with manual controls, whereas SITB integration allows automatic data synchronization.
- The application’s impact after 2 months of treatment is unknown. More research is needed to improve ongoing application quality.
- Application function was developed by continuously updating TB/health information, by tracking patients, especially patients who had moved to other health facilities, and through chat rooms and dialogues containing persuasive messages.

## Conclusions

This research improves TB medication safety through mobile application development. It gives contributions to patients, staff, policymakers, and academics.

## Supporting information

S1 File Resume of qualitative analysis (1st phase of research). (PDF)
