# Supplementary material for: Mobile application development for improving medication safety in tuberculosis patients: A quasi-experimental study protocol
Source: PLoS One. 2022 Sep 7;17(9):e0272616. doi: 10.1371/journal.pone.0272616 (PMC9451058; doi:10.1371/journal.pone.0272616)
Supplement: S1 File — (PDF) [file pone.0272616.s001.pdf]

RESUME OF QUALITATIVE ANALYSIS (1st PHASE)
(INFORMANT: CLINICAL PHARMACOLOGY SPECIALIST)

No	Question	coding	theming
1	What are the factors that affect medication adherence of tuberculosis patients? (patients, officers, health services, social environment, drug, and monitoring factors)	Patient perception of treatment	patient factors
		Age	
		Female is more vulnerable	
		Patient and family income	
		knowledge	
		education	
		Healing motivation	
		Patients are embarrassed to seek treatment	
		Drug stock	health care factors
		Service attitude	officer factor
		educate in easy language	
		side effects information	
		social environment	social factors
		stigma in society	
		side effects of the drug	drug factor
2	What are the factors that affect side effects of treatment? (Patients, officers, health services, social environment, drug, and monitoring factors)	financial condition	patient factors
		the patient did not report to the officer	
		education	
		knowledge	
		Counseling	officer factor
3	What are the factors that affect monitoring TB patients' treatment? (Patients, officers, health services, social environment, drug, and monitoring factors)	Educate the patients	officer factor
		provide information to the drug swallowing supervisor	
4	Do you think other factors affect the safety of TB medication?	already covered all	none
5	How to improve the TB medication safety?	patient understanding	patient factors
		DOT TB unit in the health facility	healthcare factors

		access to standard laboratories	
		officer understanding	officer factor
		psychosocial support	social factors
6	What are the obstacles to monitoring the safety of TB medicine?	psychological response	patient factors
		officer discipline	officer factor

QUALITATIVE ANALYSIS RESUME
(INFORMANT: POLICYMAKER)

No	Question	Coding	Theming
1	What are the factors that affect medication adherence of tuberculosis patients? (Patients, officers, health services, social environment, drug, and monitoring factors)	Motivation	patient factors
		Perception	
		Diagnosis of TB (Drug sensitive or resistant)	
		Adaptability of the patient	
		Behavior change	
		Medication reminder	
		Desire to recover	
		The boredom of the patient in taking medications	
		Side effects experienced	
		Transportation costs	
		distance between home and the health facility	
		Understanding the impact of irregular treatment	
		Shame on treatment	
		Family support	social factors
		Community support	
		Stigma a	
		Close people support	
		Discrimination	
		Drug stock	drug factor
		Information on how to deal with side effects	officer factor
		Information on how to take the drug	
		Effective communication for TB education	
		Effective media about TB information	
		Monitoring at various levels	health system factors
		The existence of monitoring tool, including Tuberculosis Information System	
		Only some health facilities provide TB programs according to standards	healthcare factors

2	What are the factors that affect side effects of treatment? (Patients, officers, health services, social environment, drug, and monitoring factors)	patient awareness	
		comorbidities in patients	
		awareness of people around the patient	
		trust between patient and officer	
		Relation between patients and officer	
		Ease of access to health workers	
		Information about handling side effects	
		Risk management system in health facilities	
3	What are the factors that affect monitoring TB patients' treatment? (Patients, officers, health services, social environment, drug, and monitoring factors)	the patient feels well	Patient factors
		the patient needs to be heard	
		communities of patients	social factors
		Family support	
		Health promotion	officer factor
		Promotion media	
		Monitoring of medication adherence virtually	
		Psychologist counseling for patients undergoing long treatment	
		Officer reminds the patient	
		Record card about control and taking medications schedule	health facility factors
		Laboratory facilities	
4	Do you think other factors affect the safety of TB medication?	stress	patient factors
		the patient does not feel alone	
		psychological support by the officer	officer factor
		health promotion	
		monitoring of taking medications virtually	
		rewards for compliant patients	
		counseling by officers	
		a stressed patient companion	social factors
5	How to improve TB medication safety?	continuous quality evaluation	health facility factors
		application to monitor patients	
6	What programs are carried out by ministries, health offices, and health centers to improve the safety of TB medication?	Clear job description	health facility factors
		risk management	
		success indicators	
		application for treatment monitoring integrated with Information System of Tuberculosis	
7	What are the	Some cadres are elderly	social factors
		Motivation from the family	

	obstacles to monitoring the safety of TB medicine?	stigma about TB is still great	
		Boredom of the patient	patient factors
		Tools for self-health monitoring	healthcare factors
8	What kind of mobile application can help improve medication safety?	Feature to evaluate health progression	self-monitoring tools
		Chat room between TB patients	interactive with TB patient community

QUALITATIVE ANALYSIS RESUME
(INFORMANT: OFFICER)

No	Question	coding	theming
1	What are the factors that affect medication adherence of tuberculosis patients? (Patients, officers, health services, social environment, drug, and monitoring factors)	patient commitment	patient factors
		motivation to heal	
		Difficulty to consume the drug	
		Nausea	
		Counseling	officer factor
		Officer reminds the patient	
		Building patient and officer trust	
		Provision of information to the drug swallowing supervisor	
		Patient commitments at the beginning of treatment	
		Explaining the impact of taking irregular medications	
		Handling side effects	
		Educate patients about side effects	
		Easier TB services for patients	health care factors
		Drug stock	drug factor
		TB treatment duration	
		Side effects	
		Coordinate with citizens	faktor eksternl
		Family responsibility to support patients	social factors
		Drug swallowing supervisor assists the patient	
		Drug swallowing supervisor understands the treatment	
		Friends who do not support TB treatment	
		Family support improve patient compliance	
2	What are the factors that affect side effects of treatment? (Patients, officers, health services, social environment, drug, and monitoring factors)	The patient cannot distinguish between side effects and clinical conditions	patient factors
		Patient understanding	
		Clear education about side effects	officer factor
		Easy communication	
		Communication for reporting side effects	
		Explain examples of side effects	
		Asking the patient about side effects during control	
		using WA to communicate regarding side effects	

3	What are the factors that affect monitoring TB patients' treatment? (Patients, officers, health services, social environment, drug, and monitoring factors)	difficulty expectorate sputum	patient factors
		working patients	
		reminding the schedule of the sputum test	officer factor
		providing motivation to the patient	
		providing education to patients	
		counseling	
		informing the treatment plan at the beginning of treatment	
		reminds sputum test schedule	
		services that make it easier for patients	healthcare factors
		use of TB information system to view sputum checking schedules	
4	Do you think other factors affect the safety of TB medication?	Provide contact number to report side effects	officer factor
		Double duty	
		The role of supportive cadres	social factors
5	How to improve TB medication safety?	patient forgets to swallow the drug	patient factors
		use medication reminder	
		Stress	
		patients have difficulty expectorate sputum	
		notifying treatment is necessary to prevent transmission	officer factor
		familial approach	
		the role of cadres is to track patients who do not regularly take medications	social factors
6	What are the obstacles to monitoring the safety of TB medicine?	working patient e.g., night shift	patient factors
		the patient buys the drug himself if he experiences side effects of the drug	
		the patient went to another health facility but did not inform of being TB treatment	
		out-of-town patients	
		patients move	
		the patient does not have a cellphone	
		family delivers phlegm	social factors
		the activeness of cadres to track incompliance patients	
		request patient contact	officer factor
7	What kind of mobile application can help improve medication safety?	monitoring patients	remote patient monitoring
		dashboard	

		reminder or alarm	reminder
		education about food consumption	educational tools
		easy-to-use application for patients	easy to use

QUALITATIVE ANALYSIS RESUME
(INFORMANT: PATIENT)

No	Question	Coding	Theming
1	What are the factors that affect medication adherence?	working patients	patient factors
		lots of activity	
		desire to heal	
		the habit of recording schedules	
		positive thinking	
		Regular bedtime	
		family support for reminding	environmental factors (family and friends)
		emotional support of the family member	
		support from friends	
2	What are the factors that affect the re-examining of sputum on time?	phlegm is difficult to expectorate	patient factors
		the belief to be able to heal	
		officer education	officer factor
3	What have you done when experiencing side effects? What factors influence the handling of side effects?	patient knowledge	patient factors
		the patient's ability to cope with emotions in order stay calm	
		patient communication to the officer	
		The activeness of the patient for consultation	
		patient initiative to deal with side effects according to the officer's orders	
		officers provide information	officer factor
		the role of the officer is to treat according to protocol	
		emotional support from officers	
		education from officers when facing effects side	
		side effects in the first two months appear more frequent	treatment factors
		the presence of interactions between drugs	

		clear information by the officer	Officer
		emotional support from officers	
		the patient's ability to calm down	patient
5	What kind of mobile application can help improve medication safety?	add insight	knowledge enhancer
		practically can be opened anywhere	practical and easy
		identifying side effects of the drug	telehealth
		means of communication with officers	
		there is a record of taking medication	dashboard
		there is a record of the development of the disease	
		alarm	Reminder
